# Onchocerciasis Fingerprints in the Geriatric Population: Does Host Immunity Play a Role?

**DOI:** 10.3390/tropicalmed6030153

**Published:** 2021-08-19

**Authors:** Cabirou Mounchili Shintouo, Robert Adamu Shey, Tony Mets, Luc Vanhamme, Jacob Souopgui, Stephen Mbigha Ghogomu, Rose Njemini

**Affiliations:** 1 Frailty in Ageing Research Group, Vrije Universiteit Brussel, Laarbeeklaan 103, B-1090 Brussels, Belgium; Cabirou.Mounchili.Shintouo@vub.be (C.M.S.); Tony.Mets@uzbrussel.be (T.M.); 2 Department of Gerontology, Faculty of Medicine and Pharmacy, Vrije Universiteit Brussel, Laarbeeklaan 103, B-1090 Brussels, Belgium; 3 Department of Biochemistry and Molecular Biology, Faculty of Science, University of Buea, Buea P.O. Box 63, Cameroon; sheynce@gmail.com (R.A.S.); stephen.ghogomu@ubuea.cm (S.M.G.); 4 Department of Molecular Biology, Institute of Biology and Molecular Medicine, IBMM, Université Libre de Bruxelles, Gosselies Campus, 126040 Gosselies, Belgium; Luc.Vanhamme@ulb.be (L.V.); jsouopgu@ulb.ac.be (J.S.)

**Keywords:** onchocerciasis, immunosenescence, immune dysfunction, elderly

## Abstract

One of the most debilitating consequences of aging is the progressive decline in immune function, known as immunosenescence. This phenomenon is characterized by a shift in T-cell phenotypes, with a manifest decrease of naive T-cells—dealing with newly encountered antigens—and a concomitant accumulation of senescent and regulatory T-cells, leading to a greater risk of morbidity and mortality in older subjects. Additionally, with aging, several studies have unequivocally revealed an increase in the prevalence of onchocerciasis infection. Most lymphatic complications, skin and eye lesions due to onchocerciasis are more frequent among the elderly population. While the reasons for increased susceptibility to onchocerciasis with age are likely to be multi-factorial, age-associated immune dysfunction could play a key role in the onset and progression of the disease. On the other hand, there is a growing consensus that infection with onchocerciasis may evoke deleterious effects on the host’s immunity and exacerbate immune dysfunction. Indeed, *Onchocerca volvulus* has been reported to counteract the immune responses of the host through molecular mimicry by impairing T-cell activation and interfering with the processing of antigens. Moreover, reports indicate impaired cellular and humoral immune responses even to non-parasite antigens in onchocerciasis patients. This diminished protective response may intensify the immunosenescence outcomes, with a consequent vulnerability of those affected to additional diseases. Taken together, this review is aimed at contributing to a better understanding of the immunological and potential pathological mechanisms of onchocerciasis in the older population.

## 1. Introduction

The elderly population, particularly the group over 85 years, is growing very rapidly. By 2050, for the first time in human history, the elderly are expected to comprise more than one-fifth of the world’s population [1]. Even more alarming is the case of some developing countries, where the older population is increasing three times faster than the global average [2]. These demographic transformations result in an increased incidence of aging-related diseases, with many consequences, including costs for households, strains on public finances, decreasing economic growth, and increasing hospitalization. Notwithstanding, unlike in most developed countries where there is provision to take care of the elderly population, in many developing countries, the populations are aging without any safeguard measures being taken to support the older population [3]. As a result, the incidence of chronic infectious conditions—commonly encountered in older people in developing countries—has increased and is expected to rise steeply in the coming years.

Human onchocerciasis is one of the most prevalent infectious diseases occurring in developing countries [4]. It is a vector-borne parasitic neglected tropical disease caused by infection with the filarial worm *Onchocerca volvulus* (*O. volvulus*) [5]. The disease is transmitted by black flies (*Simulium*) that acquire microfilariae (larval stage 1) during a blood meal from infected humans. Within 10 to 12 days, the microfilariae mature into infective larvae (larval stage 3) and can be transmitted to humans during a subsequent blood meal, where they develop into adult worms after several months. The adult worms live in subcutaneous tissues in humans for about 15 years, with adult female worms releasing close to 1600 microfilariae daily [6,7]. Worldwide, about 20.9 million people are reported to be infected with the parasite—of which 99% live in Africa—with 14.6 million having skin disease and 1.15 million suffering from irreversible unilateral or bilateral visual loss [8]. Ivermectin is the only drug approved for mass treatment of onchocerciasis. Ivermectin enhances immune function and induces immunological processes that reduce microfilariae density [9]. This dramatic reduction of microfilariae after therapy is marked by a gradual increase in the number of circulating CD4 + T-cells, which double within 1 month after treatment and return to the pretreatment baseline level by 14 months after treatment [10]. However, the drug is not able to kill the adult worms; thus, treatment needs to be repeated for the lifespan of the adult worm, and, in some endemic regions, the disease is still persistent after many years of treatment [11].

Several studies have unequivocally revealed an increase in the prevalence of onchocerciasis infection among older people, aged 50 years and above, living in endemic regions [12,13,14,15]. Most lymphatic complications, skin and eye lesions due to onchocerciasis are more frequent among the elderly population. While the reasons for increased susceptibility to onchocerciasis with age are likely to be multi-factorial, age-associated immune dysfunction could play a key role in the onset and progression of the disease [16,17].

## 2. Immunological Features of Ageing

Ageing is accompanied by a progressive decline in immune function, referred to as immunosenescence [18,19]. At the cell level, T-cell sub-populations show major changes, resulting in a shift towards a less functional status, with a corresponding decrease in the number of naive T-cells (able to react to new challenges) and an accumulation of terminally differentiated T-cells (only reacting to one specific antigen) and senescent cells [20]. On the other hand, the thymus gland atrophies with age, leading to a significant decline in the thymic output of naïve T-cells [21,22,23]. These changes are key contributors to the process of aging and are associated with a greater risk of morbidity and mortality in older people [18]. Indeed, clinical reports indicate that the ability to mount primary immune responses against novel antigens reduces significantly with age [24], leading to a decrease in response to vaccines by elderly individuals [25,26,27] and an increase in their susceptibility to diseases [28,29,30,31]. This situation is further complicated by the aging-related accumulation of senescent T-cells that secrete pro-inflammatory substances and matrix-degrading enzymes [19,32,33], favorable for the development of diseases [34]. Age-related changes in eosinophil functional activity were observed upon the examination of eosinophil degranulation of eosinophil-derived neurotoxins when stimulated with interleukin (IL)-5. A significant decrease in the eosinophils from elderly individuals in comparison to younger individuals was observed [35]. Additionally, complement analysis revealed that an increase in age is associated with the enhanced functional activity of classical and alternative complement pathways. Also, terminal pathway components (C5, C8, and C9) increased with age, and this may be a mechanism to compensate for the low clearance of pathogens and apoptotic cells due to lower cellular immunity [36]. Furthermore, aging is associated with an increased in the function of regulatory T-cells that is aimed at fooling the immune system by producing anti-inflammatory cytokines [21]. Therefore, and more generally, immunosenescence has been associated with the increased vulnerability of older subjects to inflammation-related conditions [18].

### Immunosenescence, Inflammaging, and Immune-Risk Profile in the Elderly

Inflammaging [37,38] and immune risk profile (IRP) [39] are two recent concepts regarding immunosenescence that are increasingly being recognized to be, at least in part, the cause of increased susceptibility to morbidity and mortality in older subjects. Inflammaging refers to a chronic, low-grade, but above baseline, systemic inflammation (occurring in the absence of acute infection) that is established during physiological aging [40]. It is associated with increased plasma levels of pro-inflammatory cytokines such as tumor necrosis factor α, IL-1, and IL-6, acute phase reactants such as C reactive proteins, and soluble cytokine receptors such as the TGF-β receptor family and IL-17 receptor [41,42]. This phenomenon can be caused by a number of separate yet interconnected processes, which include: (1) an age-related buildup of senescent cells that have developed a secretory pro-inflammatory character, such as senescent innate immune cells; (2) an age-related buildup of misplaced self-molecules, such as cytoplasmic or cell-free nucleic acids, which can elicit innate immune responses by interacting with a variety of pattern recognition receptors; (3) a deficiency in central tolerance that causes self-tissue damage-induced inflammation by producing an increase in the release of self-reactive T-lymphocytes; (4) a change in the composition of the gut microbiota, which might influence inflammation based on immune system activity and lifestyle [43]. Despite its core physiological purpose as a defense mechanism against viruses or foreign substances, inflammation can be harmful to one’s health if it is sustained and protracted [44]. Accordingly, studies have shown that a heightened inflammatory state may play a central role in the pathogenesis of diseases, including Alzheimer’s disease [45], Parkinson’s disease, acute multiple sclerosis, atherosclerosis, age-related macular degeneration [46], diabetes mellitus [47], osteoporosis [48], and cancer [49,50].

On the other hand, IRP is characterized by a shift in T-cell sub-population types, manifested by an inverted CD4 +/CD8 + T-cell ratio, lower numbers, and proportions of naïve T-cells, with a concomitant increase of highly differentiated memory and senescent T-cells. In a Swedish longitudinal study involving persons older than 85 years, the best predictors of survival were reported to be the absence of inverted CD4/CD8 T-cell ratios and low counts of highly differentiated cells [51]. Additionally, IRP is strongly associated with seropositivity to chronic viral infections, particularly cytomegalovirus (CMV), probably due to the chronic antigenic load that CMV delivers to T-cells [52,53]. Indeed, age-related increases in memory CD8 + T-cells are paralleled by an increase in the proportion of CMV epitope-specific T-cells. Khan et al. [54] observed that individual CMV epitope-specific CD8 + T-cells could represent up to 23% of the total CD8+ T-cells in older adults with CMV infection. CMV-specific T-cells are highly differentiated cells marked by the lack of expression of the costimulatory receptors CD27 and CD28 [55]. CD28 expression is necessary for full cellular activation and proliferation upon T-cell stimulation, and loss of CD28 expression has a major impact on T-cell function [56]. Moreover, T-cells lacking CD28 may lead to inflammaging by producing large amounts of cytokines after stimulation [57]. In this light, the clonal expansion of CMV-specific CD8 + T-cells can exacerbate human T-cell immunosenescence and, thereby, modulate the responsiveness to diseases [58].

## 3. Immunological Responsiveness to Onchocerciasis

The protective immune response of individuals against *O. volvulus* is dependent on, amongst others, type 2 immune responses mediated by IgE and eosinophils [59,60,61]. Eosinophils have been reported to be involved in the destruction of microfilariae through an antibody-dependent mechanism [62]. The antibodies involved in this process are proposed to be of the IgG isotype [62,63], with IgG3 and IgG4 playing a protective and suppressive role, respectively [64]. These antibodies can induce efficient antibody-dependent cell-mediated cytotoxicity reactions against *O. volvulus* larval stage 3 when combined with Th1 and/or Th2 cytokines [65,66]. Another mechanism by which eosinophils could destroy *O. volvulus* constituents, particularly larval stage 3, is through complement activation [67]. However, microfilariae avoid complement attack by inducing the cleavage of the complement 3 molecule into its inactive form [68]. Moreover, once established, the host’s immune system is unable to get rid of adult worms as they are capable of modulating the immune system in a way that is beneficial to their survival [69]. Indeed, *O. volvulus* has been reported to counteract the immune responses of the host through molecular mimicry [70,71] by impairing T-cell activation [72] and interfering with the processing of antigens [73]. In this light, there is growing evidence for the involvement of antigen specific regulatory T-cells (Tr1/Th3)—that produce anti-inflammatory cytokines, including IL-10 and transforming growth factor-β [74]—in the evasion of host immune responses by *O. volvulus.* Steel et al. [75] observed that the microfilariae release proteins that cause strong upregulation of cytotoxic T-lymphocyte-associated protein 4, which bind to CD80 on antigen-presenting cells and generate IL-10. IL-10 suppresses the Th1-immune response, thereby promoting chronic onchocerciasis [76,77].

On the other hand, higher frequencies of memory CD4 + T-cells were observed in onchocerciasis individuals with a corresponding upregulation of Th2-related (IL-4, STAT6, and IL-13) and Th17-related (IL-17, IL-1b, IL-6, and IL-22) genes [78]. Noteworthy, when peripheral blood mononuclear cells from onchocerciasis patients were stimulated with an *O. volvulus* antigen, they produced significantly less interferon-gamma (IFN-γ) and IL-5 compared to healthy controls [79]. IFN-γ is one of the Th1 cytokines that are reported to be involved in cellular responses to *Onchocerca* antigens; it is an aid in protection against onchocerciasis [80]. Likewise, IL-5 has been shown to have a protective role in BALB/cBYJ mice when vaccinated against *O. volvulus* infective third-stage larvae [59]. Moreover, the lifespan of microfilariae is between 12–18 months [81], and their death usually results in severe inflammation, which is responsible for most of the disease’s symptoms [82,83]. This diminished protective response, in conjunction with a heightened liberation of pro-inflammatory cytokines [84,85], may foster chronic low-grade inflammation with a consequence vulnerability of those affected to additional diseases and poor vaccine responses. In this perspective, reports indicate impaired cellular and humoral immune responses even to non-parasite antigens in onchocerciasis patients [86,87]. Poor immunogenicity of the tetanus vaccine was observed in onchocerciasis patients [86,87,88]. Cooper et al. [86] demonstrated that concurrent infection with *O. volvulus* can reduce the immune response to an unrelated antigen—such as the tetanus toxoid—by a mechanism that is suggested to involve IL-10. Furthermore, the efficacy of BCG and rubella vaccinations were significantly lower in children infected with onchocerciasis [89], thereby reflecting the clinical severity of the disease.

## 4. Immunological Markers of Onchocerciasis

The Ov16 test is the only serological test that is used for the assessment of human onchocerciasis. However, it has a moderate sensitivity of 60–80% [90]. Therefore, biomarkers that can reliably identify patients with onchocerciasis are urgently needed to ensure effective disease management [91]. The host response to different *O. volvulus* antigens represents opportunities to measure immunological markers that have diagnostic or prognostic potential, although they may have to be interpreted in the context of the protein that is being investigated. For example, Lagatie et al. [92], upon identification of three immunodominant motifs scattered over the *O. volvulus* proteome, revealed that linear epitopes from these motifs have an atypical isotype profile that is dominated by IgG1, IgG3, IgE, and IgM. This isotype profile contradicts sharply with the response against other *O. volvulus* antigens (e.g., Ov-11, Ov-16, Ov-27, Ov-29, Ov-33) or crude extracts of *O. volvulus*, which are characterized by significantly higher levels of IgG4 antibodies [64,93,94].

## 5. Clinical Spectrum of Onchocerciasis and Ageing

*O. volvulus* infection has been reported in all age groups, from children younger than 10 years to adults over 50 years of age. Although not always consistent, reports have generally revealed an increase in the prevalence of onchocerciasis with increasing age (see Table 1) [12,13,14,15,95,96,97,98,99,100], and the duration of stay in an onchocerciasis-endemic community is thought to be one of the main predictors of onchocercal infection [13]. This may be ascribed to the fact that the rate of larval stage 3 infecting the human host is greater than the rate of death of the adult worms [13,15,95,96,98,101]. Hence, there is a continuous buildup of infection since the adult worms can stay for several years in the human host. In this light, Dana et al. [13] reported that individuals who lived in an onchocerciasis-endemic community for over 60 years were at almost 6 times higher risk of *O. volvulus* infection than individuals who had stayed in the community for less than 10 years. In addition, Dozie et al. [15] reported that the increase in the prevalence of the disease with advancing age may be due to the cumulative nature of infection of the parasite acquired early in life. Notwithstanding, the elderly population may be the most infected group because aging itself is accompanied by immune dysfunction. With aging, although there is a normal antibody response to recall antigens, the ability to manufacture high-affinity antibodies that can protect elderly individuals from infection wanes. Older persons not only create lower titers of antibodies, but they also create antibodies that demonstrate diminished functionalities (e.g., neutralizing and opsonizing activities) in comparison to those produced by younger persons [102,103]. This age-associated immune dysfunction contributes to the decline of protective responses by aged individuals against infections, with a consequent increased susceptibility to diseases [104,105,106].

### 5.1. Onchocerciasis and Susceptibility of Older Persons to Diseases

As mentioned above, infection with *O. volvulus* affects the host’s resistance to other diseases, resulting in a reduced life expectancy of the host [74,107,108]. If exposed to HIV, onchocerciasis patients have a greater likelihood of becoming HIV-positive than non-onchocerciasis individuals [109]. Additionally, a report has revealed that glaucoma patients have a higher prevalence of onchocerciasis compared to individuals that are not affected by glaucoma [110]. Furthermore, onchocerciasis patients are more susceptible to epilepsy [111,112]. The more pronounced susceptibility to diseases observed in onchocerciasis infected geriatric persons could be due to the cumulative effect of immunosuppression as a result of *Onchocerca* infection and age-related immunosenescence.

### 5.2. Lymphatic Complications Due to Onchocerciasis in the Elderly Population

*O. volvulus* obstructs lymph nodes and promotes microfilariae penetration, leading to lymphadenitis. In the preliminary stage, there is atrophy of germinal centers and sinus histiocytosis and, later, the replacement of lymphoid tissue by fibro-adipose tissue, which has a perivascular pattern in the later stages [113]. Lymphatic complications as a result of onchocerciasis are more distributed among the older population than the younger population [12,15,95,100,114] (see Table 2). Furthermore, some of the lymphatic complications predispose onchocerciasis elderly patients to other forms of lymphatic complications. For example, the development of hanging groin predisposes the individual to hernia [115].

### 5.3. Onchocercal Skin Lesions among the Elderly Population

Onchocercal skin lesions (OSLs) have been reported to increase with a corresponding increase with age [117,118] (see Table 3). OSLs are associated with negative psychosocial, physical, and economic burdens. Hence, they seriously affect the quality of life, causing the stigmatization of the affected persons and a decrease in human development [119,120]. There is a spectrum of immune responses to infection, whereby some patients show minimal immune response to *O. volvulus* antigens, allowing the development of microfilariae without the presence of clinical symptoms. On the other hand, some patients have an intact and symptomatic immune response to *O. volvulus* antigens [82]. Moreover, contrary to most phenotypes of OSL [14,15,95,114,121], pruritus has been reported to be more prevalent in the younger age group [12,15,100]. This may be due to the fact that the first visible symptom of onchocerciasis is OSLs, which usually begin with intense itching, followed by irritating papular rashes [122]. The rate of acute papular onchodermatitis has been reported to increase until about 30 years; it decreases steadily with age [15,99,100,116], suggesting that acute papular onchodermatitis is common in early infections and disappears in long-standing infections, in which many microfilariae may have died [99]. From an immunological point of view, it can also be inferred that *O. volvulus* and the human immune system may have attained a state of equilibrium in the case of the elderly, which may decrease the cases of acute irritation [99].

### 5.4. Visual Impairment and Blindness

*Onchocerca volvulus* causes visual impairment, which often advances to blindness. It is the second leading infectious cause of blindness globally [123,124]. Blindness is the most severe complication of the disease and may affect more than 30% of the adult population in hyperendemic communities [117,125]. The development of keratitis depends on antigen-specific T-cell and antibody responses that control the sequence of molecular and cellular events leading to the migration of inflammatory cells to the cornea, with a consequent loss of corneal clarity [126]. Additionally, there is an upregulation of mRNA for IL-4 and IL-5 in the corneas of mice immunized with *O. volvulus* antigens upon intrastromal challenges. These cytokines were reported to regulate the severity of keratitis through the infiltration of inflammatory cells, particularly eosinophils, into the cornea [127,128,129]. Visual impairment and eye itching have been reported among several age groups, from children less than 10 years to a peak in old adults above 50 years [12,15,95,130]. Blindness is more prevalent in the elderly population [12,130,131], with some studies reporting all cases of blindness coming from the aged population [15,116,121] (see Table 4). Disability due to severe visual impairment or blindness affects productivity and effectiveness in labor and has important socio-economic implications [132]. The high frequency of blindness among the aged population may be due to the fact that they were already blind as a result of onchocerciasis before the commencement of treatment programs for the disease in their communities [121]. In addition, blindness may result from long-standing infection owing partly to the refusal of ivermectin treatment for fear of severe adverse events or persistent non-adherence and non-compliance to ivermectin intake [133,134]. A long-term impact evaluation of the African Programme for Onchocerciasis Control operations revealed that 400,000 persons—with an additional 200,000 at-risk persons—were protected from visual loss [135].

## 6. Concluding Remarks and Future Perspectives

The immune system of the elderly population is marked by the paradox of immunosenescence and inflammaging, which embody two sides of the same coin, resulting in immune disorder [137]. With aging, though there is a normal antibody response to recall antigens, the ability to manufacture high-affinity antibodies that can protect elderly individuals from infection wanes. Older persons not only create lower titers of antibodies, but they also create antibodies that demonstrate diminished functionalities [102,103]. This age-associated immune dysfunction contributes to the decline of protective responses by aged individuals against *Onchocerca* infection, which leads to the increased prevalence of onchocerciasis in elderly individuals. Th1 cytokines decrease in aged onchocerciasis patients; meanwhile, Th2 cytokines, including IL-10, increase. The production of high levels of IL-10 in older onchocerciasis patients suppresses Th1-type immunity and, thereby, favors the manifestation of chronic onchocerciasis [76,77]. Notably, most of the immunological changes observed during *Onchocerca* infections are similar to changes that occur in the immune system during aging. Therefore, it is plausible that *Onchocerca* infection in elderly individuals can aggravate the effects of immunosenescence in this population due to the synergistic immunomodulatory effects of each of them. In this perspective, it would be worthwhile to give greater attention to the prevention, diagnosis, and treatment of onchocerciasis in elderly individuals. The development of new and appropriate tools to completely eliminate the disease is most worthy of further work, as this might reverse the burden of onchocerciasis in the vulnerable geriatric population.

## Figures and Tables

**Table 1:** Distribution of onchocerciasis in the older population.

Total Number of Infected Participants	Number of Elderly Individuals Infected (%)	Age Range of Elderly Individuals	Remark per the Other Age Groups	Geographical Distribution	Authors
188	100 (53.2)	≥50	Most infected group	Nigeria	Anosike et al. [12]
99	40 (40.4)	≥55	Most infected group	Ethiopia	Dana et al. [13]
833	135 (16.2)	≥55	Overall increase with age	Ethiopia	Dori et al. [14]
889	363 (53.9)	≥50	Most infected group	Nigeria	Dozie et al. [15]
170	39 (22.9)	≥56	Most infected group	Nigeria	Kamalu and Uwakwe [95]
305	95 (31.1)	≥50	Most infected group	Cameroon	Kamga et al. [96]
122	53 (43.4)	≥50	Most infected group	Nigeria	Onekutu et al. [97]
398	145 (36.4)	>50	Most infected group	Nigeria	Opara and Fagbemi [98]
281	53 (18.9)	≥51	Overall increase with age	Nigeria	Okoye et al. [99]
3257	351 (10.8)	≥55	Most infected group	Nigeria	Murdoch et al. [100]

**Table 2:** Distribution of lymphatic complications in the older population.

Category	Number of Participants Affected	Number of Elderly Individuals Infected (%)	Age Range of Elderly Individuals	Remark per the Other Age Groups	Authors
Hernia	2	2 (100.0)	≥50	Only group affected	Anosike et al. [12]
	88	27 (30.7)	≥50	Most affected group	Dozie et al. [15]
Hanging groin	41	29 (70.7)	≥50	Most affected group	Dozie et al. [15]
	13	7 (53.8)	≥56	Most affected group	Kamalu and Uwakwe [95]
	95	43 (45.3)	≥55	Most affected group	Murdoch et al. [100]
	1	1 (100.0)	≥50	Most affected group	Okoro et al. [114]
Lymphadenopathy	73	28 (38.4)	≥50	Most affected group	Dozie et al. [15]
	6	5 (83.3)	≥50	Most affected group	Mbanefo et al. [116]
Lymphoedema	58	44 (75.9)	≥50	Most affected group	Dozie et al. [15]
Scrotal elephantiasis	18	15 (83.3)	≥50	Most affected group	Anosike et al. [12]

**Table 3:** Distribution of onchocercal skin lesions in the older population.

Category	Number of Participants Affected	Number of Elderly Individuals Infected (%)	Age Range of Elderly Individuals	Remark per the Other Age Groups	Authors
Skin depigmentation	71	50 (70.4)	≥50	Most affected group	Anosike et al. [12]
	357	201 (56.3)	≥50	Most affected group	Dozie et al. [15]
	525	109 (20.8)	≥51	Most affected group	Okoye et al. [99]
	261	91 (34.9)	≥55	Most affected group	Murdoch et al. [100]
Lichenified onchodermatitis	73	38 (52.1)	≥50	Most affected group	Dozie et al. [15]
	410	100 (24.4)	≥51	Most affected group	Okoye et al. [99]
Leopard skin	11	9 (81.8)	≥50	Most affected group	Anosike et al. [12]
	90	23 (25.6)	≥56	Most affected group	Kamalu and Uwakwe [95]
	24	8 (33.3)	≥50	Most affected group	Okoro et al. [114]
	2	2 (100)	≥50	Only group affected	Sufi and Zainab [121]
Lizard skin	8	7 (87.5)	≥50	Most affected group	Anosike et al. [12]
	68	22 (32.4)	≥56	Most affected group	Kamalu and Uwakwe [95]
Atrophy	40	30 (75.0)	≥50	Most affected group	Anosike et al. [12]
	104	35 (33.7)	≥50	Most affected group	Dozie et al. [15]
	184	45 (24.5)	≥51	Most affected group	Okoye et al. [99]
Nodule	11	6 (54.5)	≥50	Most affected group	Anosike et al. [12]
	675	178 (26.4)	≥50	Most affected group	Dozie et al. [15]
	70	21 (30.0)	≥56	Most affected group	Kamalu and Uwakwe [95]
	127	47 (37.0)	≥50	Most affected group	Kamga et al. [96]
	6	3 (50.0)	≥50	Most affected group	Okoro et al. [114]
	86	35 (40.6)	≥50	Most affected group	Mbanefo et al. [116]
	5	4 (80)	≥50	Most affected group	Sufi and Zainab [121]
Onchocercal skin disease	293	54 (18.4)	≥55	Most affected group	Dori et al. [14]
Pruritus	49	9 (18.4)	≥50	Least affected group	Anosike et al. [12]
	574	50 (8.7)	≥50	Least affected group	Dozie et al. [15]
	645	28 (4.3)	≥55	Least affected group	Murdoch et al. [100]
	105	35 (33.3)	≥50	Most affected group	Mbanefo et al. [116]
Chronic papular onchodermatitis	349	84 (24.1)	≥50	Third most affected group	Dozie et al. [15]
	1034	187 (18.1)	≥51	Fourth most affected group	Okoye et al. [99]
	155	33 (22.3)	≥55	Most affected group	Murdoch et al. [100]
Acute papular onchodermatitis	273	29 (10.6)	≥50	Fourth most affected group	Dozie et al. [15]
	576	37 (6.4)	≥51	Least affected group	Okoye et al. [99]
	233	18 (7.7)	≥55	Third most affected group	Murdoch et al. [100]
	76	11 (14.5)	≥50	Fourth most affected group	Mbanefo et al. [116]

**Table 4:** Distribution of ocular lesions in the older population.

Category	Number of Participants Affected	Number of Elderly Individuals Infected (%)	Age Range of Elderly Individuals	Remark per the Other Age Groups	Authors
Ocular lesions	16	13 (81.3)	≥50	Most affected group	Anosike et al. [12]
	506	268 (53.0)	≥50	Most affected group	Kirkwood et al. [130]
Eye itching	14	5 (35.7)	≥50	Most affected group	Anosike et al. [12]
	297	80 (26.9)	≥50	Second most affected group	Dozie et al. [15]
Impaired vision	1009	452 (44.8)	≥50	Most affected group	Dozie et al. [15]
	76	22 (28.9)	≥56	Most affected group	Kamalu and Uwakwe [95]
	77	14 (18.2)	≥50	Fourth most affected group	Okoro et al. [114]
	23	19 (82.6)	≥50	Most affected group	Mbanefo et al. [116]
	462	76 (16.5)	≥50	Second most affected group	Akogun [131]
Anterior uveitis	282	81 (28.7)	≥50	Most affected group	Dozie et al. [15]
Punctate opacity	241	79 (32.8)	≥50	Most affected group	Dozie et al. [15]
	197	54 (27.4)	≥50	Most affected group	Akogun [131]
Sclerosing keratitis	232	93 (40.1)	≥50	Most affected group	Dozie et al. [15]
	247	59 (23.9)	≥50	Most affected group	Akogun [131]
Blindness	18	15 (83.3)	≥50	Most affected group	Anosike et al. [12]
	6	6 (100.0)	≥50	Only group affected	Dozie et al. [15]
	179	51 (28.5)	≥50	Second most affected group	Okoro et al. [114]
	1	1 (100.0)	≥50	Only group affected	Mbanefo et al. [116]
	2	2 (100.0)	≥50	Only group affected	Sufi and Zainab [121]
	434	246 (56.7)	≥50	Most affected group	Kirkwood et al. [130]
	339	101 (29.8)	≥50	Most affected group	Akogun [131]
	98	76 (77.6)	≥50	Most affected group	Schwartz et al. [136]

## Data Availability

Not applicable.
